# Epidermal CD147 expression plays a key role in IL-22-induced psoriatic dermatitis

**DOI:** 10.1038/srep44172

**Published:** 2017-03-08

**Authors:** Cong Peng, ShengXi Zhang, Li Lei, Xu Zhang, Xuekun Jia, Zhongling Luo, Xiaoyan Huang, Yanhong Kuang, Weiqi Zeng, Juan Su, Xiang Chen

**Affiliations:** 1 Department of Dermatology, Xiangya Hospital, Central South University, Changsha, Hunan, China; 2 Hunan Key Laboratory of Skin Cancer and Psoriasis, Xiangya Hospital, Central South University, Changsha, Hunan, China

## Abstract

Psoriasis is a chronic inflammatory skin disease characterized by abnormal keratinocyte proliferation and terminal differentiation. Interleukin-22 (IL-22) and the transcription factor Stat3 play pivotal roles in the pathogenesis of psoriasis. CD147 is a transmembrane glycosylation protein that belongs to the immunoglobulin superfamily. Our previous studies have shown that CD147 is a marker of high keratinocyte proliferation and poor keratinocyte differentiation as well as a psoriasis susceptibility gene. The current study demonstrates that CD147 is highly expressed in psoriatic skin lesions. Specific CD147 over-expression in the epidermis of K5-promoter transgenic mice promotes imiquimod (IMQ)-induced psoriasis-like inflammation characterized by acanthosis, granular layer loss and inflammatory cell infiltration. We also found that IL-22 increases CD147 transcription *in vitro* and *in vivo* and that Stat3 binds directly to the CD147 promoter between positions −854 and −440, suggesting that CD147 expression is up-regulated in patients with psoriasis through Stat3 activation. In addition, CD147 knockdown dramatically blocks IL-22-mediated Stat3 activation as well as IL-22-induced cytokine, chemokine and antimicrobial factor expression. Together, these findings show that CD147 is a novel and key mediator of IL-22-induced psoriatic alterations in the epidermis and might be a therapeutic target in patients with psoriasis.

Psoriasis is an immune-mediated chronic inflammatory skin disease characterized by abnormal epidermal keratinocyte hyperplasia and differentiation as well as T cell, neutrophil and macrophage infiltration^1^,^2^,^3^,^4^. Psoriatic skin lesions are typically characterized by the redness, thickness and scaling induced by epidermal hyperproliferation and inflammation. These symptoms severely impair patient quality of life.

The epidermis is composed of stratified squamous epithelium, and it protects the body from environmental agents and prevents dehydration^5^,^6^. Epidermal keratinocytes play critical roles in the pathogenesis of psoriasis. Various inflammatory cytokines, chemokines and growth factors can be strongly induced in the keratinocytes of psoriatic lesions, causing inflammation, epithelial hyperplasia and immune cell mobilization, ultimately resulting in chronic skin lesion formation^7^,^8^.

Although the etiology and pathogenic mechanisms underlying psoriasis remain unclear, accumulating evidence suggests that Th17 and Th22 cells and their associated cytokines play important roles in the pathology of psoriasis^9^,^10^,^11^. Interleukin-22 (IL-22), a cytokine that belongs to the IL-10 family, is primarily produced by immune cells^12^. The IL-22 receptor is expressed on epithelial cells and stromal cells instead of immune cells^13^,^14^, indicating that IL-22 exclusively targets epithelial cells and contributes to inflammation. Recent studies have shown that IL-22 is involved in many inflammatory diseases including psoriasis. One study showed that IL-22 levels in both the psoriatic lesions and sera of patients with psoriasis were significantly higher than those in the skin and sera of control participants^15^,^16^,^17^. In addition, IL-22 activates the JAK/STAT3 signaling pathway^18^, induces the expression of proinflammatory and antimicrobial molecules such as S100A8 in human keratinocytesand promotes hyperproliferation and inhibits differentiation in keratinocytes^12^,^19^,^20^,^21^.

CD147, also named with Basigin or EMMPRIN (EMMPRIN, extracellular matrix metalloproteinase), is a transmembrane glycosylation protein that belongs to the immunoglobulin superfamily. It is involved in various biological processes such as T cell maturation, spermatogenesis, embryo implantation and retinal development^22^,^23^,^24^,^25^. Our previous studies have shown that CD147 is a molecular marker of high keratinocyte proliferation and low keratinocyte differentiation as well as a psoriasis susceptibility gene^26^. CD147 is highly expressed in the PBMCs of patients with psoriasis^27^.

The current study showed that CD147 expression was dramatically increased in human psoriatic skin lesions as well as in imiquimod (IMQ)-induced psoriasis-like skin lesions in a mouse model. In addition, CD147 promoted IMQ-induced psoriasis in transgenic mice over-expressing KC-specific CD147 (K5-CD147). We also showed that IL-22 increased the expression of CD147 at the transcriptional level as well as the expression of the transcriptional factor Stat3, which binds directly to the CD147 promoter and likely plays a major role in CD147 over-expression in human psoriatic lesion skins.

## Materials and Methods

### Reagents and antibodies

Chemical reagents including Tris, NaCl, and SDS, which were used for molecular biology experiments and buffer preparation, were purchased from Sigma-Aldrich (St. Louis, MO, USA). Cell culture media and other supplements were purchased from Life Technologies, Inc. (Rockville, MD, USA). GAPDH antibodies (Proteintech, USA) were diluted at 1:8,000; CD147 antibodies (SC-13976, epitope for 1–200 amino acids of human CD147 (Santa Cruz, CA, USA) were diluted at 1:500; and p-Stat3 (#9145, Cell Signaling Technology, Danvers, MA), p-Stat1(#9167, Cell Signaling Technology, Danvers, MA) and Stat3(#9139, Cell Signaling Technology, Danvers, MA) antibodies (Cell Signaling Technology, Danvers, MA) were diluted 1:500.

### Expression vector construction

*CD147* promoters were amplified via polymerase chain reaction (PCR) using HeLa cell genomic DNA. The PCR products were purified using a Cycle Pure Kit (Sangon Inc. Shanghai, China) and sub-cloned into pGL3-basic plasmids (Promega, WI, USA) using KpnI and Hindlll enzymes.

### Cell culture and transfection

HEK293T cells maintained in our lab were grown in Dulbecco’s modified Eagle medium (DMEM, Thermo Scientific, MA, USA) supplemented with 10% fetal bovine serum (FBS) and antibiotics. HaCaT cells were cultured in DMEM (Hyclone, USA) supplemented with 10% FBS (Bioind, Israel) in a humidified atmosphere with 5% CO_2_ at 37 °C. Normal human epidermal keratinocytes (NHEKs) were obtained from the circumcision skin tissue samples of volunteers (n = 10) who provided written informed consent for their samples to be used. The NHEKs were cultured in serum-free Keratinocyte Growth Medium 2 (PromoCell, German) for at least 5 days. All cells were cultured at 37 °C in a humidified atmosphere with 5% CO_2_. For the transfection experiments, the cells were transfected with different plasmids using TurboFect Transfection Reagent (Thermo Scientific, MA, USA). The reagent and DNA were diluted in Opti-MEM (Invitrogen, CA, USA) and incubated for 15 min. The mixture was subsequently added to the cells growing in plates, which were incubated for 36 to 48 h to facilitate transfection.

### Keratinocyte isolation and culture

Child foreskin was incubated in dispase II overnight at 4 °C. The epidermis was gently scraped and washed using cold PBS, and the epidermal cells were separated following incubation with Trypsin (0.05%) at 37 °C for 10 min. Digestion was neutralized with 5% FBS medium. After being washed twice with serum-free Keratinocyte Growth Medium 2 (PromoCell, German), the cell pellet was re-suspended in 10 ml of serum-free Keratinocyte Growth Medium and cultured.

### Reverse-transcription real time PCR

Total RNA was extracted with Trizol (Invitrogen), and cDNA was synthesized via reverse transcription using a Script cDNA Synthesis Kit (Bio-Red). RT-qPCR was performed using an UltraSYBR Mixture with ROX (CWBio, Beijing, China) according to the manufacturer’s instructions on an ABI 7500 fluorescence quantitative PCR instrument (Applied Biosystems, USA). The reaction mixture contained 0.5 ml of forward and reverse human or mouse primers, as described in Tables 1 and 2. All values were normalized to *GAPDH*. All reactions were conducted in triplicate across at least three independent experiments. Relative quantification was performed using the ΔΔC_T_ method, and the results were expressed in linear form using the formula 2^−ΔΔCT^. The results were considered as significant when a difference in expression of 2-fold or more was detected.

### Western blotting

Proteins from cell lysates were denatured in SDS and separated with 10% SDS–PAGE before being transferred onto PVDF membranes (Millipore, USA). Nonspecific binding was blocked using 5% nonfat milk in TBST (10 mM Tris–HCl, 150 mM NaCl, 0.05% Tween 20, pH 7.5) for 1 h at room temperature. The membranes were incubated with specific antibodies against β-actin (diluted at 1:1,000, Santa Cruz, CA, USA), CD147 (diluted at 1:1,000, Abcam, Cambridge, UK), STAT1, STAT3, phospho-STAT1, and phospho-STAT3 (diluted at 1:1,000, Cell Signaling Technology, MA, USA) overnight. The membranes were washed with TBST and then incubated with a secondary HRP-conjugated goat anti-mouse or anti-rabbit IgG antibody (diluted at 1:5,000, Santa Cruz, CA, USA).

### Human skin samples

This study was reviewed and approved by local ethics Institutional Review Board (IRB) (Xiangya Hospital, Central South University, IRB-201512526). All experiments were conducted in accordance with the Declaration of Helsinki Principles. Written informed consent was obtained for all procedures. Biopsies were taken from the lower backs of healthy volunteers and from the psoriatic plaques of patients with psoriasis and frozen at −80 °C. The skin lesion biopsy samples were compared with the healthy skin biopsy samples.

### Immunohistochemical analysis

The slides were subjected to immunohistochemical staining, which was performed according to the manufacturer’s recommendations. Briefly, the slides were baked at 60 °C for 2 h, dewaxed in turpentine, rehydrated in a graded ethanol series, and then treated with 3% H_2_O_2_ for 10 min to inhibit endogenous peroxidases. The slides were then pretreated in a microwave oven (in 0.01 M sodium citrate buffer, pH 6.0) for 5 min, blocked with 10% goat serum for 30 min and then incubated with CD147 antibodies (1:100, Santa Cruz, CA) overnight at 4 °C in a humidified chamber. The slides were washed the next day and incubated with the appropriate secondary antibody (anti-rabbit 1:200, Santa Cruz, CA) for 1 h. Horseradish peroxidase-streptavidin (Santa Cruz Biotechnology, Santa Cruz, CA, USA) was added to the slides. The samples were stained for 5 min with a 0.05% 3,3′-diaminobenzidine substrate and then counterstained with hematoxylin for 5 min before being mounted in neutral balsam.

### Immunohistochemical evaluation

Two investigators who were blinded to the identities of the immunohistological sections performed the immunohistochemical evaluation. Three fields from each sample were randomly selected and viewed under a microscope at a magnification of 400 × . Staining intensity was graded on a scale of 0 to 3 as follows: negative, 0: weakly positive, 1:moderately positive, 2; strongly positive. Staining percentage was scored as follows: 0:none; 1:1–20%, 2:25–50%, 3: more than 60%. The immunoreactivity intensity distribution index (IRIDI) was calculated by multiplying the two scores together to determine the final positive staining rate of each section. Samples that were unrecognizable or heavily covered by melanin were excluded from analysis.

### Luciferase assay

HEK293T cells were transfected with *CD147 promoter-Luc, SV-40-Renilla-Luc* or *Stat3* in the presence of transfection reagent. Exactly 30 h after transfection, the cells were disrupted using a passive lysis buffer, and the cell lysates were analyzed for Firefly and Renilla luciferase activity using a Dual-luciferase Assay Kit (Promega, Madison, WI, USA).

### Chromatin immunoprecipitation assay

A ChIP assay was performed using a Chromatin Immunoprecipitation Kit (Millipore, MN). Briefly, the cells were collected and crosslinked with 1% (v/v) formaldehyde for 10 min at room temperature. Nuclei were subsequently isolated via the lysis of the cytoplasmic fraction, and the chromatin was divided into 150–900 bp fragments via ultrasonic disruption using a standard microtip and a Branson W250D Sonifier (four pulses, 60% amplitude, duty cycle 40%). The sonicated nuclear fractions were divided for input control and incubated at 4 °C overnight with anti-Stat3 antibodies (Cell Signaling Technology, MA, USA) or control IgG antibodies (Cell Signaling Technology, MA). After incubating with 30 ml of ChIP grade protein A/G-agarose beads for 2 h at 4 °C, the antibody-protein-DNA complexes were eluted from the beads and digested using Proteinase K (40 mg) for 2 h at 65 °C, followed by spin column-based DNA purification. Finally, the genomic DNA recovered from the ChIP assays was amplified via qPCR with primers () specific for the Stat3 binding region of the CD147 promoter. The specificity of each primer set was verified by an analysis of the dissociation curve of each gene-specific PCR product.

### ELISA

The kerationcytes with knock down of CD147 expression was treated with IL-22 for 12 hr, and then, the supernatant was collected and centrifuged at 1000*g for 20 min following instruction. ELISA kits were used to detect CXCL1 (cat. no. SEA041Hu, Cloud-Clone Corp., Wuhan, China), CXCL3 (cat. no. SEB604Hu, Cloud-Clone Corp., Wuhan, China), and TNF-α (cat. no. SEA133Hu, Shanghai ExCell Biology, Inc., Shanghai, China) concentrations according to instructions and the results had been normalizated.

### Generation of transgenic mice specifically over-expressing CD147 in epithelial cells

The animal study protocol was approved by the Ethics Committee of Xiangya Hospital (Central South University, China, #2015110134). Shanghai Biomodel Organism Science and Technology Development Co (Shanghai, China) generated transgenic mice specifically over-expressing CD147 in epithelial cells. Briefly, CD147 genomic DNA fragments were amplified from mouse genomic DNA and sub-cloned into pCDNA3.1(−) hygromycin-CAG vectors containing a K5 promoter. These vectors were microinjected as transgenes into fertilized C57BL/6J mouse oocytes, and transgenic offspring were screened via PCR using the indicated left (TCAGCAACCTTGACGTAAATGTT) and right primers (CTTGTCATCATCGTCCTTGTAGTC). Two of five founders were selected for additional breeding and experiments according to the CD147 expression levels in their skin. Neither strain of CD147 transgenic mice exhibited any baseline skin phenotypes for at least 6 months.

### IL-22 induces epidermal changes in mice

All procedures involving animals were performed in accordance with the institutional animal welfare guidelines of Central South University and approved by the Ethics Committee of Xiangya Hospital (#2015100231). C57BL/6 J mice were purchased from the Department of Laboratory Animal Medicine of Central South University and used at 4–6 weeks of age. Twenty-five microliters of PBS, either alone or containing 500 μg of recombinant IL-22 (Prospec, Israel), was intradermally injected into the ears of anesthetized mice using a microinjector once every two days. This procedure was repeated 4 or 8 times. Five mice in each experimental group were used, and each experiment was repeated at least three times. After injection, the mouse ears were collected and immediately frozen in liquid nitrogen for RNA quantification and 10% neutral formalin solution for hematoxylin and eosin (H&E) staining and immunohistochemical staining analysis.

### IMQ induces psoriasis-like inflammation in a mouse model

The animal study was approved by the Ethics Committee of Xiangya Hospital (Central South University, China). Four- to 6-week-old mice were treated with daily topical doses of 62.5 mg of IMQ cream (5%, 3.125 mg of the active compound; Aldara, 3 M Pharmaceuticals), which was applied to their shaved backs for 6 consecutive days as described previously^28^. Control mice were treated in a similar manner with a vehicle cream.

### Analysis of psoriasis-like inflammation scores pertaining to the lesions on the backs of mice

A scoring system based on the clinical Psoriasis Area and Severity Index (PASI) was used to evaluate the skin inflammation on the backs of mice as described previously^29^. Briefly, erythema, scaling, and thickening were graded on a scale from 0 to 4 as follows: 0, none; 1, slight; 2, moderate; 3, marked; and 4, very marked. The level of erythema was scored using a table with red taints. The cumulative score (erythema plus scaling plus thickening) served as a measure of inflammation severity (scale: 0–12).

### Suspension of single cells isolated from the backs of mice and FACS analysis

Skin samples from the backs of IMQ-treated or untreated mice were dissected and incubated at 37 °C overnight in 1 U/ml dispase II (Roche) plus 1.0 mg/ml collagenase (Sigma-Aldrich, St. Louis, MO). After 2 h, enzyme activity was stopped using 10% FBS medium, and the mixtures were filtered with 40-mm nylon filters to obtain single-cell suspensions. For FACS staining, 1 million cells were incubated with 1.0 mg/ml GR-1 (conjugated with PE; BioLegend). A post-acquisition analysis was performed using FlowJo software (Tree Star).

#### Statistical analyses

All statistical analyses were performed using GraphPad Prism 6 (GraphPad Software, San Diego, CA, USA). All data are presented as the mean ± SD. One-way ANOVAs and two-tailed *S*tudent’s t-tests were used to evaluate significance. P-values < 0.05 were considered as significant, and P-values < 0.01 were considered as highly significant.

## Results

### CD147 over-expression in the epidermis of patients with psoriasis and a mouse model of psoriasis-like skin lesions

**Figure 1:**
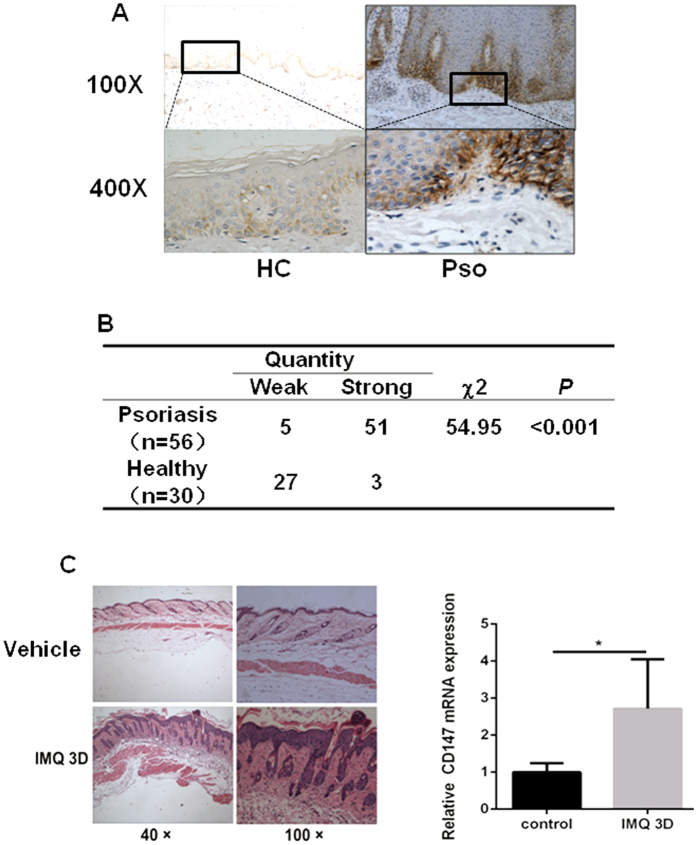
Increased epidermal CD147 expression in psoriasis. (**A**) Representative images of the IHC analyses of CD147 expression in the skin of a patient with psoriasis (Pso) and a healthy control (Hc). (**B**) Statistical analyses of CD147 expression in the skin samples of Psos and Hcs via IHC. (**C**) CD147 expression levels in the epidermis of IMQ-treated mice were assessed via qPCR. Significant differences were evaluated using a two-way ANOVA, and the asterisk (*) indicates a significant difference (n = 5, p < 0.05).

To investigate the involvement of CD147 in psoriasis, we analyzed its expression in psoriatic human skin samples using immunohistochemistry (IHC). As shown in Fig. 1A and B, CD147 expression was significantly up-regulated in the epidermis of patients with psoriasis compared with that of healthy controls and the distribution of CD147 is mainly in cellular membrane which is consistent with previous report. Negative control was shown in supplementary Figure 1 indicated that CD147 staining is specific for its expression in Fig. 1. In addition, CD147 expression is clearly limited to the basal layer of the epidermis and does not occur in other layers (Fig. 1A,B), suggesting that CD147 plays a key role in initiating keratinocyte proliferation in psoriasis. Topical IMQ administration induced psoriasis-like skin lesions characterized by erythema, skin thickening, scaling, epidermal alterations (acanthosis and parakeratosis) and angiogenesis as well as T cell and neutrophil infiltration (Fig. 1C) in mice as previously described^12^,^19^. Regarding the mouse model, our qPCR results showed that CD147 expression was clearly increased in the aforementioned psoriasis-like skin lesions on the backs of mice (Fig. 1D). These results demonstrate that CD147 expression is significantly increased in IMQ-treated mice and the skin lesions of patients with psoriasis, suggesting that CD147 expression in psoriatic epidermal cells is functionally involved in the pathogenesis of psoriasis.

### Specific CD147 over-expression in the epidermis promotes skin lesions in a mouse model of IMQ-induced psoriasis

**Figure 2:**
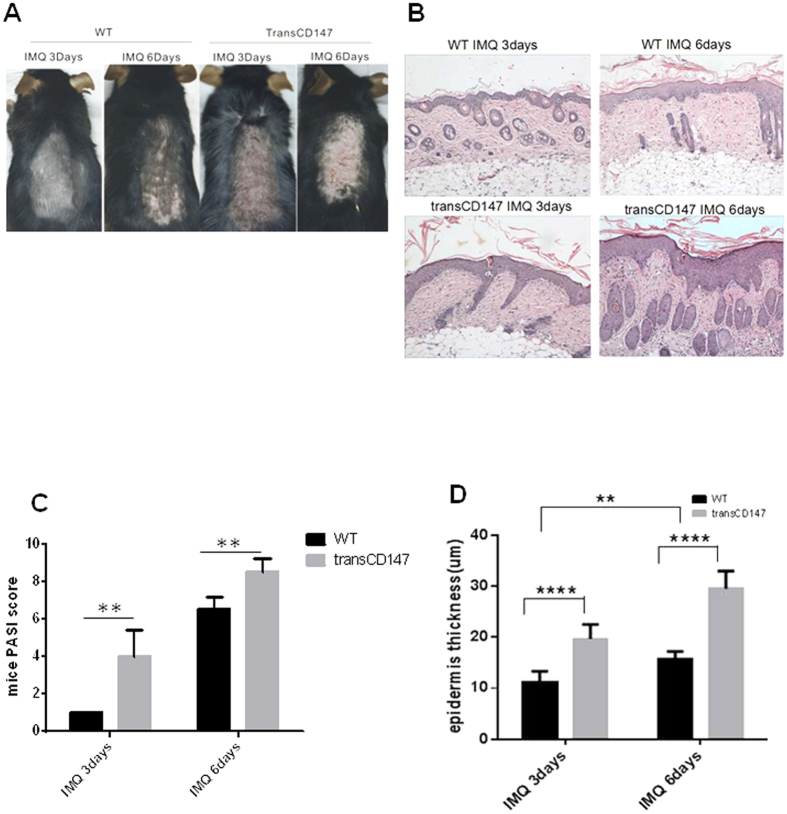
Specific CD147 over-expression in the epidermis promotes psoriasis-like lesions induced by IMQ. (**A**) IMQ was applied topically to K5-CD147 and WT mice daily. After 3 or 6 days of treatment, the mice were photographed and sacrificed for skin lesion analysis. Macroscopic phenotypical representations of psoriasis-like skin lesions in K5-CD147 or WT mice (one representative mouse is presented, n = 4). (**B**) H&E staining showing the skin on the backs of K5-CD147 and WT mice after IMQ treatment (original magnification 100x). One representative picture is shown for each treatment regimen. (**C**) PASI scores were used to assess skin lesions in K5-CD147 or WT mice after IMQ treatment. Significant differences were evaluated using a two-way ANOVA, *n = 4, p < 0.05. (**D**) Epidermal thickness measurements were performed at the indicated time points. Significant differences were evaluated using a two-way ANOVA, and the asterisk (*) indicates a significant difference (n = 4, p < 0.05).

**Figure 3:**
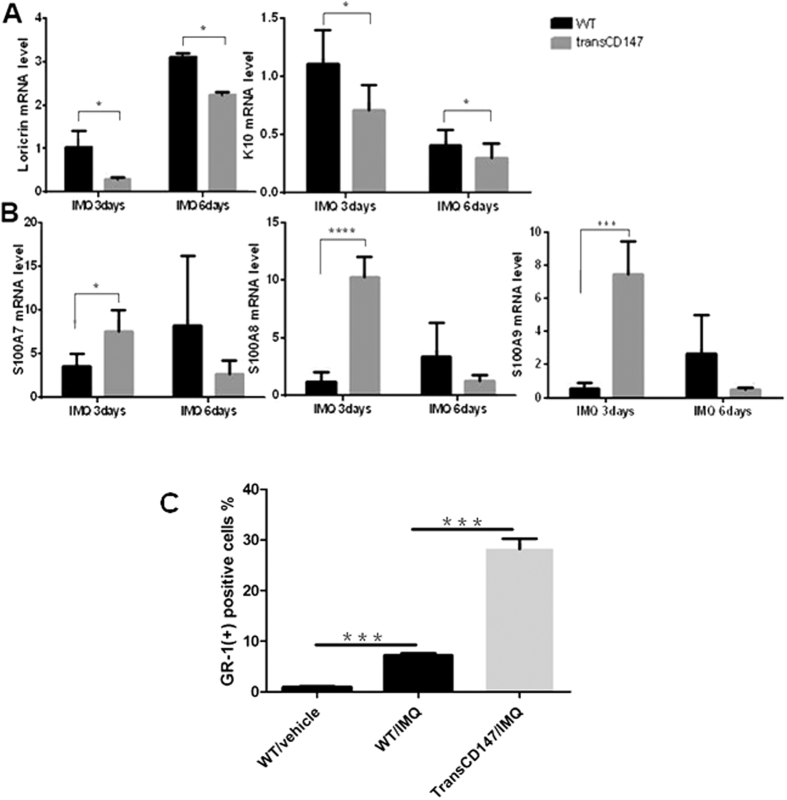
CD147 over-expression in the epidermis affects keratinocyte cellular function and the inflammatory response after IMQ treatment. (**A**,**B**) IMQ was applied topically to K5-CD147 and WT mice daily. After 3 or 6 days of treatment, RNA was isolated from the skin on the backs of both strains of mice, and an RT-qPCR analysis was performed to assess the expression of the indicated genes. Significant differences were evaluated using a two-way ANOVA, n = 4, p < 0.05. (**C**) IMQ was applied topically to K5-CD147 and WT mice (n = 4). After 3 days of treatment, a single-cell suspension was prepared from samples of the epidermis from the backs of the mice, as described in the *Materials and Methods* section. A flow cytometry (FCM) analysis of the dermal single-cell suspensions was performed to assess GR-1 expression. The mean percentages of the positive cells from each group, which contained four mice, are indicated. Significant differences were evaluated using a two-way ANOVA, and the asterisk (*) indicates a significant difference (p < 0.05).

CD147 levels were shown to be highly elevated in the skin lesions of IMQ-treated mice and patients with psoriasis. To test whether CD147 plays a critical role in the pathogenesis of psoriasis, we genetically engineered mice possessing a K5 promoter to over-express CD147 in a keratinocyte-specific manner. After IMQ treatment, CD147 substantially increased the expression of the psoriasiform phenotype in a mouse model of IMQ-induced psoriasis (Fig. 2B). Epidermal thickness and PASI scores were significantly increased in K5-CD147 mice compared with wild-type (WT) mice (Fig. 2C,D), suggesting that CD147 expression in keratinocytes promotes epidermal hyperplasia *in vivo*. Furthermore, we found that CD147 over-expression in the epidermis dramatically decreased the expression of the terminal differentiation markers K10 and loricrin (Fig. 3A) and increases the expression of S100A7, S100A8 and S100A9 (Fig. 3B). In addition, increased GR-1-positive inflammatory cell infiltration was noted in K5-CD147 mice compared with WT mice after IMQ treatment (Fig. 3C). Together, our findings demonstrate that CD147 aggravates psoriatic inflammation by inhibiting terminal keratinocyte differentiation and promoting keratinocyte hyperplasia, immune cell infiltration and proinflammatory cytokine expression.

### IL-22 up-regulates CD147 expression in keratinocytes

**Figure 4:**
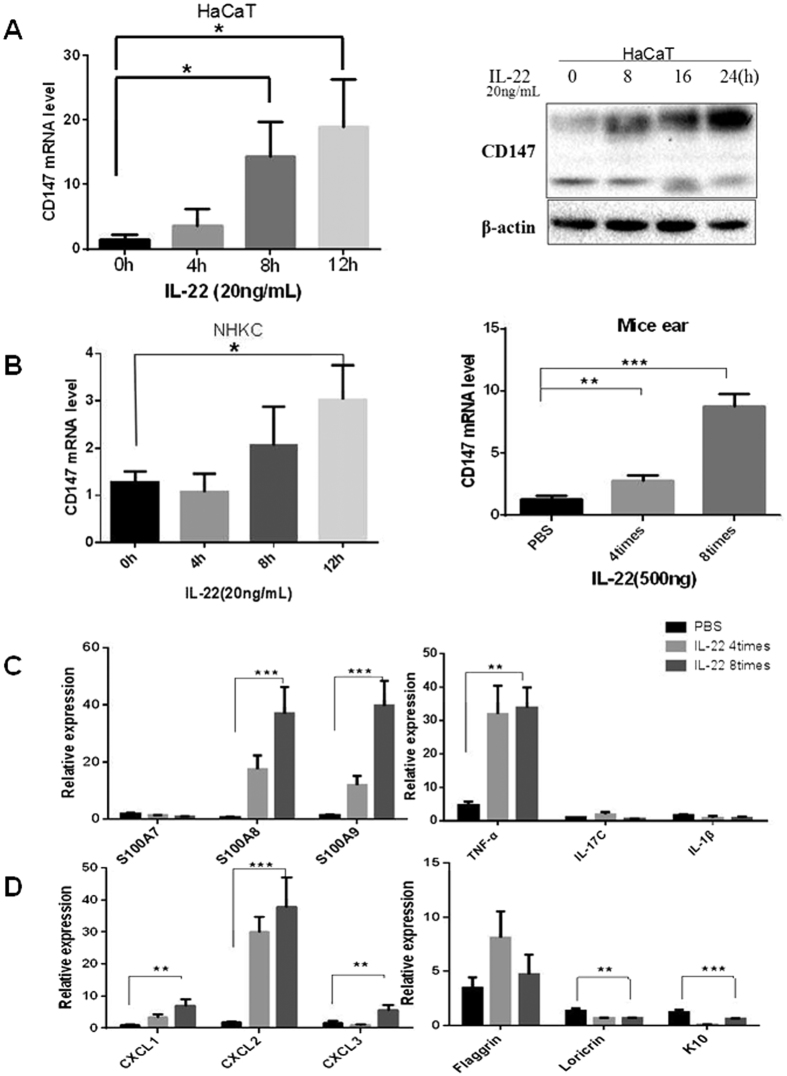
IL-22 increases CD147 in the epidermis and promotes keratinocyte cellular function. (**A**) HaCaT cells were treated with IL-22 (20 ng/ml) at different time points as indicated. RNA was extracted for RT-qPCR (left panel), and Western blotting was performed to assess CD147 protein expression (right panel). (**B**) NHEKs were isolated as described in the *Materials and Methods* section and exposed to IL-22 (20 ng/ml) at different time points as indicated. RNA was extracted for RT-qPCR (left panel). For the *in vivo* experiments, RNA was prepared from mice ears (n = 4) that were intradermally injected with PBS or IL-22 as described in the *Materials and Methods* section, and RT-qPCR was performed to assess CD147 expression (right panel) as indicated (**C**,**D**).

IL-22 is a cytokine that plays a critical role in the pathogenesis of psoriasis^20^ and is secreted by inflammatory cells such as Th1 and Th17, the latter of which has a structure similar to IL-10 but performs functions similar to IL-17^30^. Evidence indicates that IL-22 expression levels are high in the blood of patients with psoriasis and that high IL-22 expression levels are strongly correlated with disease severity^16^. This study revealed that IL-22 regulates CD147 expression in keratinocytes (see Fig. 4A and B, right panel) and that CD147 mRNA and protein levels were dramatically increased in HaCaT keratinocytes after IL-22 treatment compared with untreated cells. Furthermore, we performed *in vivo* experiments to confirm that IL-22 increases CD147 expression in keratinocytes. In these experiments, IL-22 was injected into mouse skin, and CD147 expression was examined via qPCR. CD147 expression was 2.1-fold and 6.8-fold higher in treated mice than in control mice after 4 and 8 IL-22 injections, respectively (Fig. 4B, left panel). In addition, we tested the effects of IL-22 on the inflammatory response in the mouse epidermis, namely, those on cytokine and chemokine expression. The results showed that the transcription levels of S100A8, S100A9, TNF-α, CXCL1, CXCL2, CXCL3 were up-regulated, whereas the levels of keratinocyte differentiation markers (e.g., loricrin and K10) were decreased after IL-22 treatment (Fig. 4C,D).

### IL-22 up-regulates CD147 expression through Stat3

**Figure 5:**
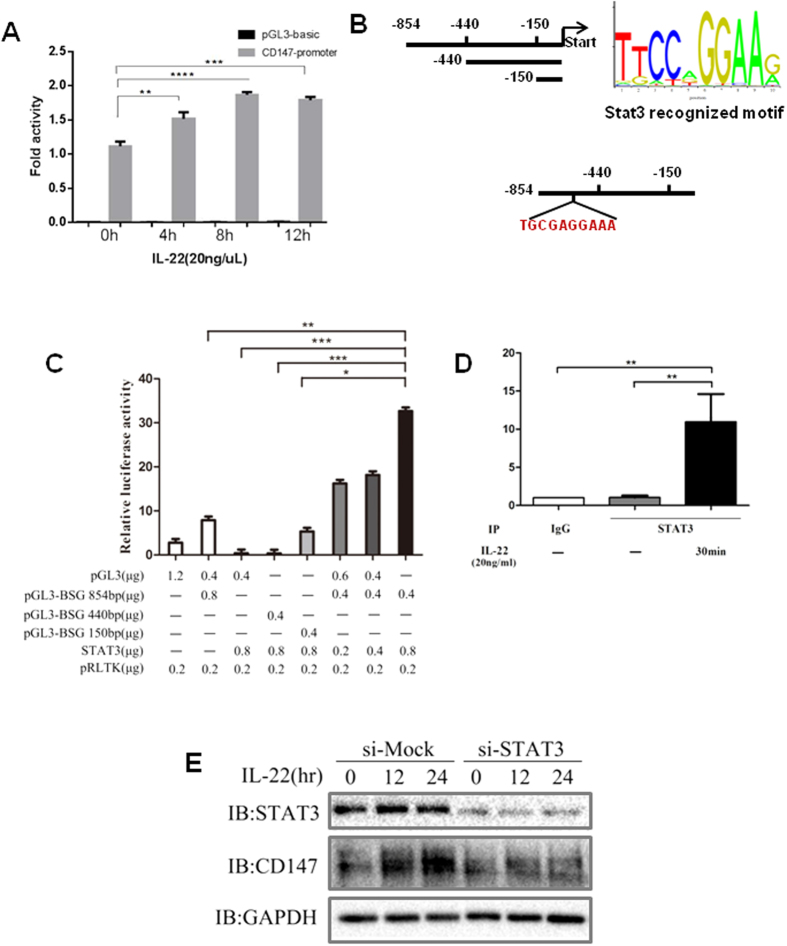
IL-22 increases CD147 expression in the epidermis and promotes keratinocyte cellular function. (**A**) HaCaT cells were co-transfected with a plasmid mixture containing the *CD147 promoter* (−*854) luciferase reporter* gene (0.4 μg) and the *Renilla luciferase* gene (0.1 μg) for normalization. After 20 h of transfection, the cells were starved for 16 h and then treated with IL-22 (20 ng/ml) for various times as indicated. *Firefly luciferase* activity was determined in the cell lysates and normalized against *Renilla luciferase* activity. Significant differences were evaluated using Student’s *t*-test, *p < 0.05. (**B**) Schematic diagram showing the fragment of the CD147 promoter in the *PGL3-luciferase* reporter gene. (**C**) Various amounts of Stat3 were transfected with a plasmid mixture containing the *truncated CD147 promoter-luciferase reporter* gene as indicated and the *Renilla luciferase* gene (20 ng) for normalization. After 24 h of transfection, *Firefly luciferase* activity was assessed in the cell lysates and normalized against *Renilla luciferase* activity. Significant differences with three times individual repeat were evaluated using Student’s *t*-test, *p < 0.05. (**D**) HaCaT cells were starved for 16 h and then treated with IL-22 (20 ng/ml) as indicated. Then, a ChIP assay was performed to examine the Stat3 recognition of the CD147 promoter as described in the *Materials and Methods* section. Significant differences with three times individual repeat were evaluated using a Student’s *t*-test, *p < 0.05. (**E**) Stat3-SiRNAs were transfected into HaCaT Keratinocytes cells. After 20 h of transfection, the cells were starved for 16 h and then treated with IL-22 (20 ng/ml) for various times as indicated. Western blotting was performed to examine protein expression, as indicated.

The aforementioned results indicate that IL-22 increases CD147 transcriptional expression in keratinocytes; therefore, we sought to determine how IL-22 regulates CD147 transcriptional expression. We generated PGL3-CD147 (-854 bp) luciferase vectors (Fig. 5B) using CD147 promoters containing -854. The results showed that IL-22 dramatically increased CD147 promoter activity (Fig. 5A). Stat3 is a critical transcriptional factor in the IL-22 signaling pathway, and it regulates a variety of target genes such as TNF-a and S100A7. To determine whether Stat3 regulates CD147 promoter activity, we co-transfected Stat3 and PGL3-CD147 (−854 bp) into HEK293T cells. The results showed that Stat3 activates the CD147 promoter (data not shown). We also constructed a fragment of the CD147 promoter (Fig. 5B, left panel) to identify the Stat3-regulated region. As shown in Fig. 5C, Stat3 dramatically increased PGL3-CD147 (−854 bp) activity but did not affect PGL3-CD147 (−440 bp) or PGL3-CD147 (−150 bp) activity, indicating that Stat3 regulates CD147 promoter activity in the region between −854 bp and −440 bp. We then analyzed this sequence (from −854 to −440 bp) using a Stat3 motif (Fig. 5B, right panel) from the *JASPAR database* and found that it contained a Stat3 binding site (score >0.85; Fig. 5B, lower panel). To confirm that Stat3 binds to the CD147 promoter, a ChIP assay was performed, and the results showed that Stat3 recognizes the CD147 promoter in HaCaT keratinocytes after IL-22 treatment. This result was not found in the group treated with the IgG control antibody or the group that did not receive treatment (Fig. 5D), suggesting that Stat3 is a key transcriptional factor in CD147 expression and that its involvement in CD147 expression is facilitated by IL-22.

### The effect of CD147 on IL-22 induced signaling pathways in keratinocytes

**Figure 6:**
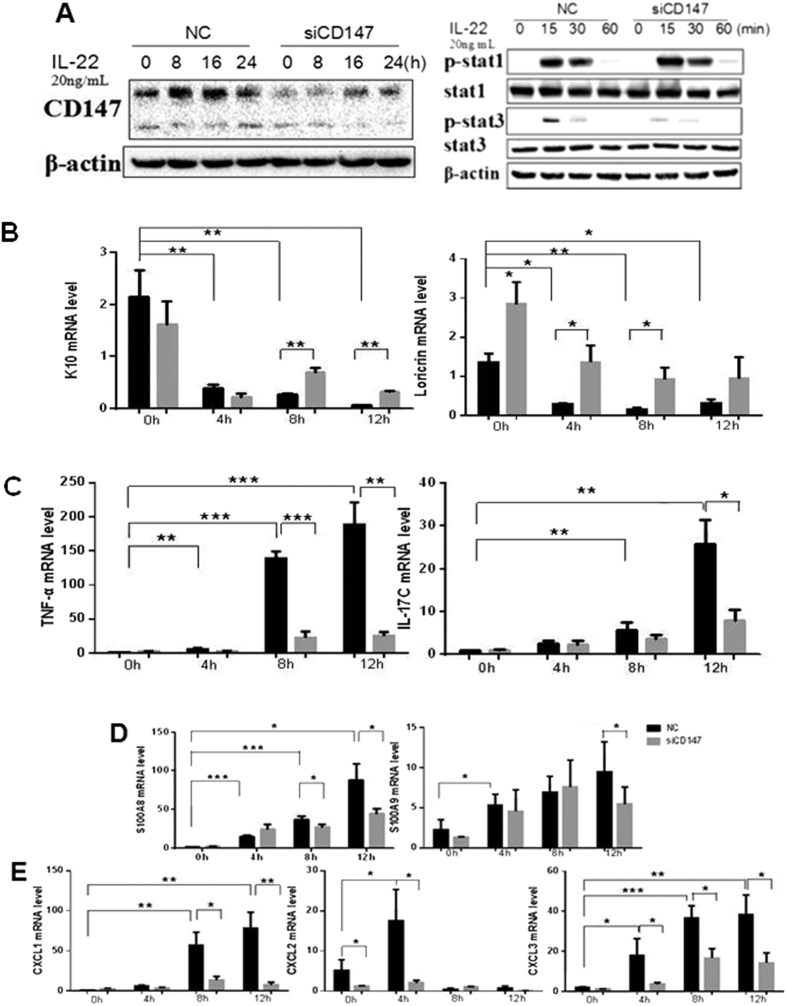
CD147 knockdown inhibits IL-22-induced cellular effects. (**A**) CD147-SiRNAs were transfected into HaCaT Keratinocytes cells. After 20 h of transfection, the cells were starved for 16 h and then treated with IL-22 (20 ng/ml) for various times as indicated. Western blotting was performed to examine protein expression, as indicated. (**B**–**E**) RNA was extracted from the CD147 knockdown cells. RT-qPCR was performed to assess gene expression as indicated.

The above results showed that CD147 over-expression in the epidermis promotes IMQ-induced psoriasis-like skin lesions in mice and that IL-22 up-regulates CD147 transcriptional expression through Stat3; therefore, we sought to determine whether CD147 controls the IL-22 induced signaling pathways in keratinocytes. To determine the influence of CD147 on IL-22-induced signaling pathways, CD147-specific siRNAs were transfected into HaCaT cells to inhibit CD147 expression (Fig. 6A). Interestingly, Stat3 phosphorylation, but not Stat1 phosphorylation, was dramatically attenuated after IL-22 treatment (Fig. 6B). In addition, we tested the effects of CD147 on IL-22 pathway target genes, as shown in Fig. 6C,D,E and supplementary Figure 2, CD147 knockdown significantly raised loricrin and K10 expression and blocked S100A8, S100A9, IL-17C, TNF-α, CXCL1, CXCL2, and CXCL3 transcriptional expression (Fig. 6C–E) as well as secretion of CXCL1, CXCL3 and TNF-a (S Fig. 2) in HaCaT keratinocytes indicating that CD147 plays a critical role in the IL-22 signaling pathway.

## Discussion

Although psoriasis is an aberrant immune disease and T cells play an important role its pathogenesis^4^,^7^,^11^, accumulating evidence suggests that psoriatic inflammation derive from epidermal epithelial cells and innate immune cells^31^,^32^,^33^. Keratinocytes affected by psoriasis not only exhibit uncontrolled proliferation but also drive the pathogenesis of the disease. Genetic alterations in the epidermis might cause skin disorders that clinically and histologically resemble human psoriasis. Over-expressed molecules in mice such as Stat3 and IL-17C in keratinocytes develop skin inflammation that exhibits the histological and molecular characteristics of psoriasis^34^,^35^. Psoriatic skin inflammation also depends on the presence of immune cells including T cells and their cytokines, indicating that psoriatic skin lesions result from interactions between immune cells and keratinocytes.

The increases in epidermal thickness associated with psoriasis are caused by basal layer dysfunction^36^,^37^,^38^. Our results demonstrate that CD147 is over-expressed in human psoriatic skin lesions. Importantly, expression of CD147 is only in basal layer of epidermis, not in other layers (Fig. 1A,B), which indicated CD147 may control keratinocyte growth in psoriasis. Consistent with these findings, CD147 expression was also increased in the IMQ-induced psoriasis-like skin lesions of mice (Fig. 1C). To investigate the role that CD147 plays in the epidermis, we generated epithelial cells containing a K5 promoter specifically over-expressing CD147. Although CD147 transgenic mice did not spontaneously develop psoriasis-like skin lesions, psoriasiform skin inflammation was promoted in these mice via IMQ treatment (Fig. 2B). At 3 and 6 days after IMQ treatment, the epidermis of the K5-CD147 mice was thicker than that of WT mice (Fig. 2D). In addition, higher PASI scores were noted in K5-CD147 mice than WT mice (Fig. 2C). The granular layer of the transgenic mice appeared to be less thick than that of the WT mice, whereas the cornified layer of the transgenic mice was more compact than that of the WT mice (Fig. 2D).

Keratinocyte terminal differentiation plays a critical role in stratum corneum formation, which involves the conversion of living keratinocytes to corneocytes. This process occurs in the granular layer and is characterized by the generation of several differentiation markers such as K10 and loricrin. CD147 over-expression in the epidermis dramatically attenuates K10 and loricrin expression in keratinocytes after IMQ treatment (Fig. 3A). These results are consistent with those of a previous study, which found that CD147 is a marker of keratinocyte hyperproliferation and poor keratinocyte differentiation^39^. In addition, S100A7, A8 and A9 expression levels were significantly increased in K5-CD147 mice after IMQ treatment compared with WT mice (Fig. 3B). Consequently, the numbers of GR-1-positive cells were dramatically increased in K5-CD147 mice (Fig. 3C). S100A family members are calcium-binding proteins produced by keratinocytes that protect the skin from microorganisms^40^,^41^ and exert pro-inflammatory effects based on the chemotactic effects of neutrophils and CD4(+) cells^42^,^43^. S100A family member expression levels are increased in psoriatic skin lesions^40^,^41^,^44^.

IL-22 is a cytokine produced by T and NK cells (but not B cells) as well as monocytes and resident keratinocytes in patients with psoriasis^15^,^20^,^45^. The major targets of IL-22 include the epithelial cells of the skin, lungs, gut and kidneys but not immune cells^46^. As previous studies have shown, IL-22 expression is significantly elevated in psoriatic skin lesions^20^,^47^. IL-22 regulates the terminal differentiation and proliferation of keratinocytes; it induces the secretion of chemokines such as CXCL1, 2, and 3, and it up-regulates the expression of antimicrobial molecules such as members of the S100A family in keratinocytes^12^,^30^. Transgenic mice over-expressing IL-22 exhibited the following psoriatic skin alterations: acanthosis, granular layer loss and cornified layer compaction^19^,^47^,^48^, whereas the IL-22-deficient mice exhibited no IMQ-induced psoriasis-like inflammation^12^. IL-22 activates the transcriptional factor Stat3, which plays a key role in the IL-22 signaling pathway^47^,^49^,^50^. Stat3 knockdown attenuates the effects of IL-22 on keratinocyte cellular functions^51^,^52^. This study found that IL-22 increases CD147 expression at both the mRNA and protein levels (Fig. 4A,B) and increases CD147 promoter activity (Fig. 5A). Furthermore, Stat3 directly recognizes its motif in the CD147 promoter, which is located between −854 and −440 bp (Fig. 5B,C). A ChIP assay confirmed that Stat3 binds to the CD147 promoter (Fig. 5D), which facilitated increases in CD147 expression in psoriasis-like skin lesions. Consistent with Stat3 regulation of CD147 expression, knock down of Stat3 dramatically decreases CD147 expression, as shown in Fig. 5E.

In addition, we found that CD147 knockdown dramatically blocked IL-22-induced cellular effects including keratinocyte terminal differentiation as well as S100A family member and cytokine or chemokine expression (Fig. 6B–D). Interestingly, CD147 knockdown significantly attenuated p-Stat3 expression (Fig. 6A), which partially explains how CD147 affects the IL-22 signaling pathway. K5-Stat3C transgenic mice that specifically express constitutively activated Stat3 in the epidermis develop psoriasis-like skin lesions, indicating that Stat3 activation is critical for psoriasis development^34^. Although the details regarding the regulation of Stat3 phosphorylation via CD147 are unclear, we noted that cavoelin-1 negatively regulates Stat3 activity by directly binding to Stat3 under conditions of psoriasis^53^. CD147 negatively regulates caveolin-1 expression and activity^29^,^54^. This finding has provided us with clues regarding the relationship between CD147 and Stat3 that we will investigate in the future.

In summary, we demonstrated that CD147 participates in the pathogenesis of chronic psoriatic inflammation. CD147 expression is significantly elevated in human psoriatic skin lesions and IMQ-induced skin lesions on the backs of mice. Specific CD147 over-expression in the epidermis promotes IMQ-induced psoriasis-like inflammation. Il-22, a critical psoriasis-related cytokine, increases CD147 expression *in vitro* and *in vivo* through Stat3. CD147 knockdown affects IL-22-induced keratinocyte terminal differentiation, cytokine/chemokine production and Stat3 activation. Taken together, our study showed that CD147 is a novel and key mediator of IL-22-induced psoriatic alterations in the epidermis and might be a therapeutic target for psoriasis.

## Additional Information

**How to cite this article**: Peng, C. *et al*. Epidermal CD147 expression plays a key role in IL-22-induced psoriatic dermatitis. *Sci. Rep.* **7**, 44172; doi: 10.1038/srep44172 (2017).

**Publisher's note:** Springer Nature remains neutral with regard to jurisdictional claims in published maps and institutional affiliations.

## Supplementary Material

Supplementary Data

## Figures and Tables

**Table 1:** Primers for human.

Gene name	Primers
GAPDH	Forward: *ctctgctcctcctgttcgac*
	Reverse: *gcccaatacgaccaaatcc*
CD147	Forward: *ccgcaaccaccttactcg*
	Reverse: *ggacagaggtttggatggtg*
S100A7	Forward: *ccaaacacacacatctcactca*
	Reverse: *tcagcttgagtgttgctcatc*
S100A8	Forward: *gccaagcctaaccgctataa*
	Reverse: *atgatgcccacggacttg*
S100A9	Forward:*gtgcgaaaagatctgcaaaa*
	Reverse: *tcagctgcttgtctgcattt*
CXCL1	Forward: *tcctgcatcccccatagtta*
	Reverse: *cttcaggaacagccaccagt*
CXCL2	Forward: *cccatggttaagaaaatcatcg*
	Reverse: *cttcaggaacagccaccaat*
CXCL3	Forward: *aaatcatcgaaaagatactgaacaag*
	Reverse: *ggtaagggcagggaccac*
IL-17C	Forward: *ccctcagctacgacccagt*
	Reverse: *cttctgtggatagcggtcct*
TNF-a	Forward: *cagcctcttctccttcctgat*
	Reverse: *gccagagggctgattagaga*
K10	Forward: *ccatcgatgaccttaaaaatcag*
	Reverse: *cgcagagctacctcattctcata*
Filaggrin	Forward: *ggactctgagaggcgatctg*
	Reverse: *tgctcccgagaagatccat*
loricrin	Forward: *ctcacccttcctggtgctt*
	Reverse: *gaggtcttcacgcagtcca*

**Table 2:** Primers for mouse.

Gene name	Primers
GAPDH	Forward: *atggtgaaggtcggtgtga*
	Reverse: *aatctccactttgccactgc*
CD147	Forward: *ccgcaaccaccttactcg*
	Reverse: *ggacagaggtttggatggtg*
S100A7	Forward: *gcctcgcttcatggacac*
	Reverse: *cggaacagctctgtgatgtagt*
S100A8	Forward: *tccttgcgatggtgataaaa*
	Reverse: *ggccagaagctctgctactc*
S100A9	Forward: *gacaccctgacaccctgag*
	Reverse: *tgagggcttcatttctcttctc*
CXCL1	Forward: *gactccagccacactccaac*
	Reverse: *tgacagcgcagctcattg*
CXCL2	Forward: *aaaatcatccaaaagatactgaacaa*
	Reverse: *ctttggttcttccgttgagg*
CXCL3	Forward: *ccccaggcttcagataatca*
	Reverse: *tctgatttagaatgcaggtcctt*
IL-17C	Forward: *cctctagctggaacacagtgc*
	Reverse: *gcggttctcatctgtgtcg*
TNF-a	Forward: *ctgtagcccacgtcgtagc*
	Reverse: *ttgagatccatgccgttg*
K10	Forward: *gaacaacttgcagaaaagaatcg*
	Reverse: *tgtggtgagttccttgctctt*
Filaggrin	Forward: *ggactctgagaggcgatctg*
	Reverse: *tgctcccgagaagatccat*
loricrin	Forward: *ggttgcaacggagacaaca*
	Reverse: *catgagaaagttaagcccatcg*
