# Supplementary material for: Epidermal CD147 expression plays a key role in IL-22-induced psoriatic dermatitis
Source: Sci Rep. 2017 Mar 8;7:44172. doi: 10.1038/srep44172 (PMC5341158; doi:10.1038/srep44172)

Supplementary Data

Epidermal CD147 expression plays a key role in IL-22-induced psoriatic dermatitis

Cong Peng[1, 2], ShengXi Zhang[1, 2], Li Lei[1, 2], Xu Zhang[1, 2], Xuekun Jia[1, 2], Zhongling Luo[1, 2], Xiaoyan Huang[1, 2], Yanhong Kuang[1, 2],Weiqi Zeng[1, 2], Juan Su[1, 2#]and Xiang Chen[1, 2#]

[1]Department of Dermatology, Xiangya Hospital, Central South University, Changsha, Hunan, China

[2]Hunan Key Laboratory of Skin Cancer and Psoriasis, Xiangya Hospital, Central South University, Changsha, Hunan, China

#Address correspondence to Xiang Chen, M.D., Ph.D., Department of Dermatology, Xiangya Hospital, Central South University, Xiangya Road #87, Changsha, Hunan, China, 410008; Tel: +86-731-84327377; Fax: +86-731-84328478;

E-mail: chenxiangck@126.com

#Address correspondence to Juan Su, M.D., Ph.D., Department of Dermatology, Xiangya Hospital, Central South University, Xiangya Road #87, Changsha, Hunan, China, 410008; Tel: +86-731-84327377; Fax: +86-731-84328478; E-mail: 540020068@qq.com

Supplementary Figure Legends

Figure 1. Staining of CD147 in healthy control and psoriasis. (A) Representative images of the IHC analyses of CD147 expression in the skin of a patient with psoriasis (Pso) and a healthy control (Hc). **(B)** Representative images of the negative control in the skin of a patient with psoriasis (Pso) and a healthy control (Hc) by IHC with incubating rabbit-IgG replace for specific secondary antibody.

Figure 2. The effect of CD147 on chemockine or cytokine secretion in heratinocytes. CD147-SiRNAs were transfected into HaCaT Keratinocytes cells. After 20 h of transfection, the cells were starved for 16 h and then treated with IL-22 (20 ng/ml) for 12 h and the supernatant was collected and centrifuged at 1000*g for 20 min following instruction. ELISA kits were used to test with indicated protein, as described in *Materials and Methods*. Significant differences with three times individual repeat were evaluated using a Student's *t*-test, *$p<0.05$.

Supplementary Figure 1

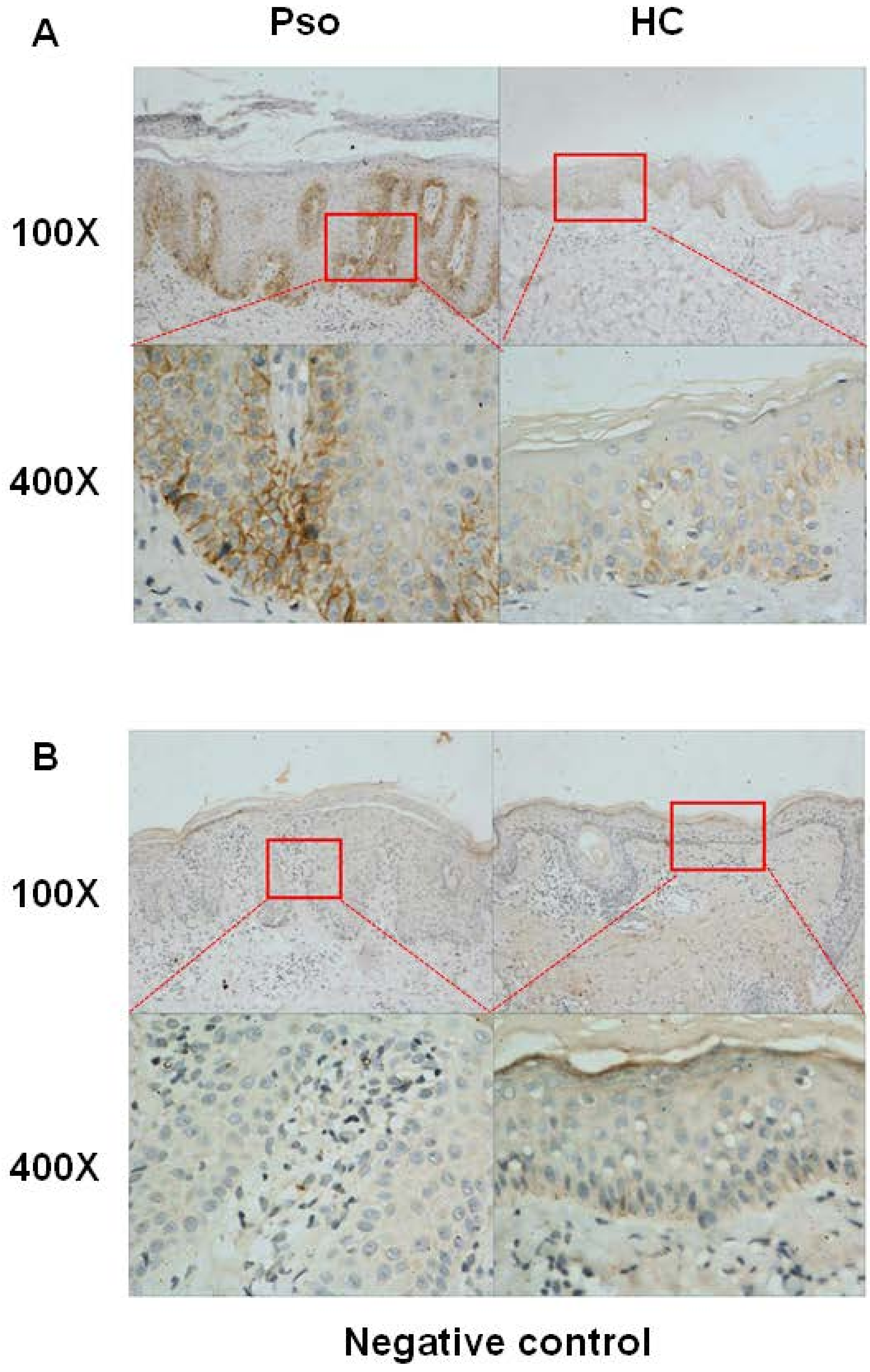

Supplementary Figure 2

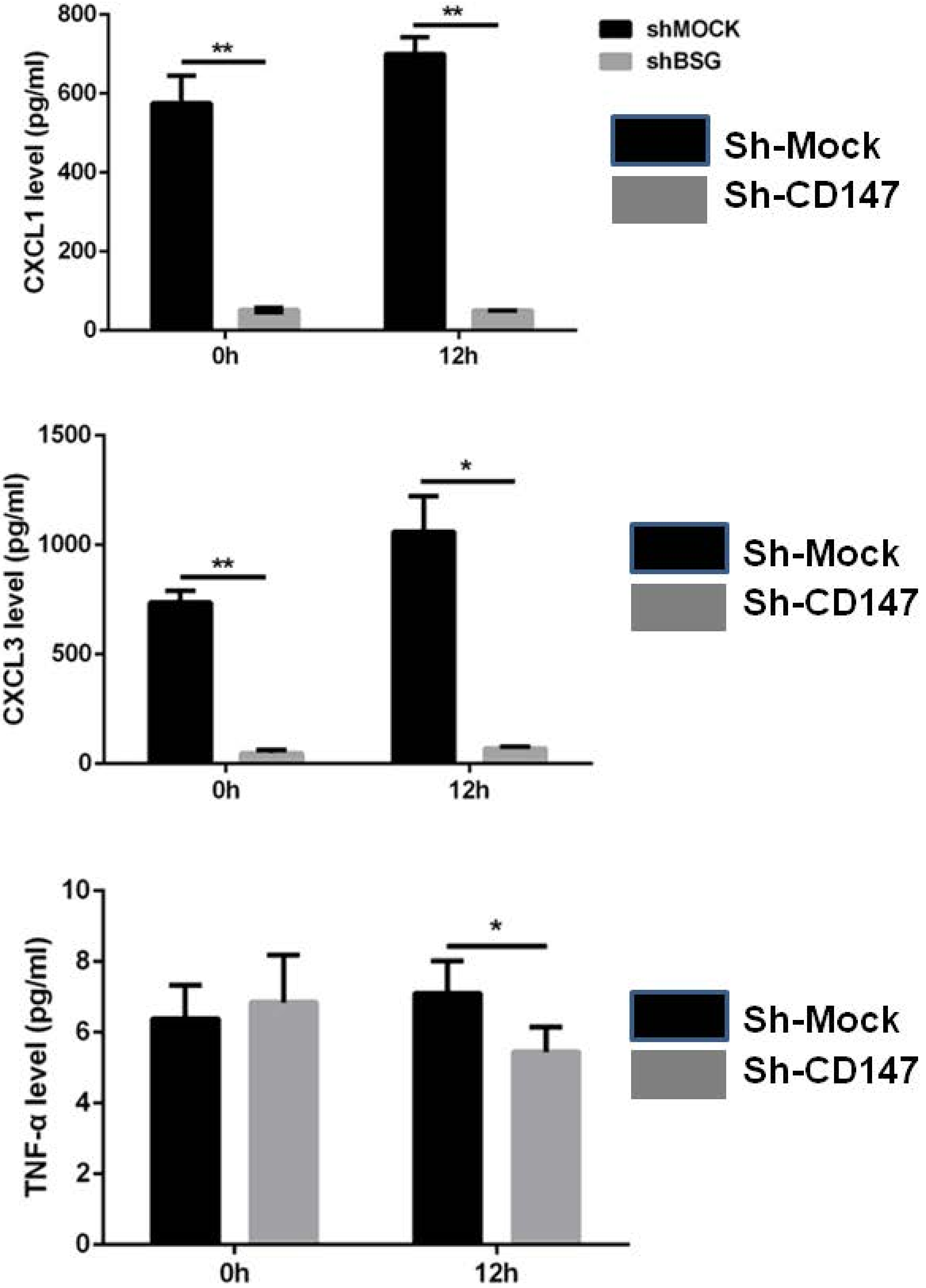

Supplement: Supplementary Data [file srep44172-s1.pdf]
